# Sensitive Determination of Trace 4-Nitrophenol in Ambient Environment Using a Glassy Carbon Electrode Modified with Formamide-Converted Nitrogen-Doped Carbon Materials

**DOI:** 10.3390/ijms232012182

**Published:** 2022-10-12

**Authors:** Bing Wang, Quanguo He, Guangli Li, Yaohang Long, Gongyou Zhang, Hongmei Liu, Jun Liu

**Affiliations:** 1 School of Biology and Engineering, Guizhou Medical University, Guiyang 550025, China; 2 Engineering Research Center of Medical Biotechnology, Guizhou Medical University, Guiyang 550025, China; 3 School of Life Science and Chemistry, Hunan University of Technology, Zhuzhou 412007, China

**Keywords:** formamide-converted, nitrogen doped carbon material, 4-nitrophenol, voltammetric detection

## Abstract

Sensing trace amounts of 4-nitrophenol (4-NP) as a harmful substance to organisms even in small quantities is of great importance. The present study includes a sensitive and selective electrochemical sensor for detecting 4-NP in natural water samples using formamide-converted nitrogen-carbon materials (shortened to f-NC) as a new material for electrode modification. The structure and morphology of the f-NC were set apart by SEM, TEM, XRD, XPS, FTIR, Raman, and the electrochemical performance of the f-NC were set apart by CV, EIS and CC. We studied the electrochemical behaviour of 4-NP on the glassy carbon electrode modified with f-NC before and after pyrolysis treatment (denoted as f-NC1/GCE and f-NC2/GCE). In 0.2 M of H_2_SO_4_ solution, the f-NC2/GCE has an apparent electrocatalytic activity to reduce 4-NP. Under the optimal conditions, the reduction peak current of 4-NP varies linearly, with its concentration in the range of 0.2 to 100 mM, and the detection limit obtained as 0.02 mM (S/N = 3). In addition, the electrochemical sensor has high selectivity, and the stability is quite good. The preparation and application of the sensor to detect 4-NP in water samples produced satisfactory results, which provides a new method for the simple, sensitive and quantitative detection of 4-NP.

## 1. Introduction

Nitroaromatic compounds in industrial production are often raw materials or intermediates of medicine, dyes, and pesticides. However, many nitroaromatic compounds, such as nitrobenzene, nitrotoluene and nitrophenol, are harmful to the environment, due to their potential to be highly toxic to humans, animals and plants. Therefore, the sensing of nitroaromatic compounds is crucial for assessing the risk of different environmental samples. In particular, acute inhalation or ingestion of 4-NP in the short term can lead to headache, drowsiness, nausea and cyanosis [1]. In addition, reports of 4-NP to be a potential carcinogen, teratogen and mutagen [2]. Its application should be strictly controlled and supervised. Therefore, developing a simple, rapid, sensitive and accurate 4-NP analysis method is necessary to detect and quantify it in different samples.

Traditional techniques for detecting 4-NP involve gas chromatography-mass spectrometry [3], high-performance liquid chromatography [4], chemiluminescence [5], capillary zone electrophoresis [6] and fluorescence [7]. Although these traditional methods can be used to detect 4-NP, the cost is high, and the operation is cumbersome, and therefore not conducive to online detection. Electrochemical methods have many advantages, such as easy operation, high sensitivity, low cost and rapid response [8,9,10,11,12]. It can overcome the shortcomings of the above traditional methods and can potentially be applied in 4-NP analysis. Recently, various types of electrochemical sensors have been used to measure 4-NP. Although many modified electrodes based on functionalised graphene oxide or graphene [13,14,15], NiO nanoparticle/ionic liquid [16], α-MnO_2_ wrapped MWCNTs [17], PdAg nanoparticle infused metal-organic framework [18] and carbon nanofiber decorated with Sm_2_O_3_ nanoparticles [19] have been described for the determination of 4-NP successfully. Challenges remain in developing new materials with simple preparation and excellent electrocatalytic properties for 4-NP electrochemical sensors.

At present, nitrogen-doped carbon materials have attracted a lot of attention. The introduction of nitrogen into carbon materials has many advantages. On the one hand, the good bonding between carbon and nitrogen greatly changes the electronic properties of carbon materials, and we significantly improved the electronic conductivity. On the other hand, the doping of nitrogen causes many lattice defects in carbon materials, which can function as the active sites for the catalytic reaction. At present, there are two main methods for the synthesis of nitrogen-doped carbon materials, in-situ doping and post-treatment. In-situ doping introduces a special precursor with nitrogen atoms in the synthesis process of carbon materials. After polymerisation and carbonisation, nitrogen atoms enter the carbon materials. Commonly used nitrogen-containing precursors include polyimides [20], ethylenediamine [21], ionic liquids [22], melamine [23], polypyrrole [24], acetonitrile [25], urea [26] and nitrogen-containing metal-organic framework compounds (MOFs) [27]. The post-treatment is used to modify the pre-synthesised carbon materials by nitrogen-rich substances as nitrogen sources. However, the nitrogen content in most nitrogen-doped carbon materials is relatively low, and the synthesis steps are cumbersome.

In this paper, we have developed a simple and sensitive method for 4-NP detection using a new nitrogen-doped carbon material, which is prepared by condensation and carbonisation of formamide (F.A.). F.A. is cheap and low toxic, containing both a primary amino group and a carbonyl group. The C-N bond can be established using the Schiff base reaction, thus forming one-dimensional molecular chains, and the nitrogen content of the formamide-converted nitrogen-doped carbon materials is up to 9.48 wt%. The prepared f-NC before and after pyrolysis treatment (shortened to f-NC1 and f-NC2, respectively) was modified on the surface of glassy carbon electrode (GCE) and used for 4-NP analysis. The best reduction peak current intensity of f-NC2/GCE significantly increased compared with bare GCE, and GCE changed with f-NC1 (f-NC1/GCE). Finally, the application of f-NC2/GCE for the sensitive, rapid and accurate determination of 4-NP in environmental water samples and the modified electrode had high stability and good reproducibility.

## 2. Results and Discussion

### 2.1. Morphological and Structure Characterisation

**Figure 1:**
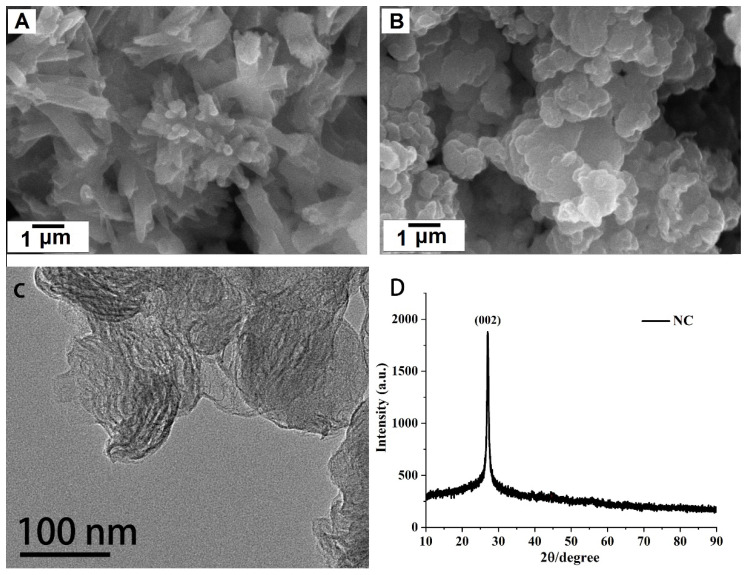
SEM images of f-NC1 (**A**) and f-NC2 (**B**); TEM image of f-NC2 (**C**) and XRD pattern of f-NC2 (**D**).

SEM images of f-NC material before and after pyrolysis (f-NC1 and f-NC2) are presented in Figure 1. Figure 1A shows the morphology of f-NC1, and strip-shaped sheets with a smooth surface can be observed. After high-temperature carbonisation, the typical morphology of the material changed significantly. A lamellar structure similar to graphene is formed (Figure 1B). The wrinkle and overlapping structure of f-NC2 were helpful to increase the specific surface area. Its particular structure provides plentiful active sites for 4-NP reduction. Figure 1C presents the TEM image of the f-NC2. Two-dimensional wrinkled and overlapping thin nanosheets can be observed. The XRD result of the f-NC2 is shown in Figure 1D. The diffraction peak at 2θ = 26.6° was sharp, matching with an elevation of (002), the typical peak of carbon nitride phase, indicating that high purity f-NC2 with good crystallinity was successfully prepared.

**Figure 2:**
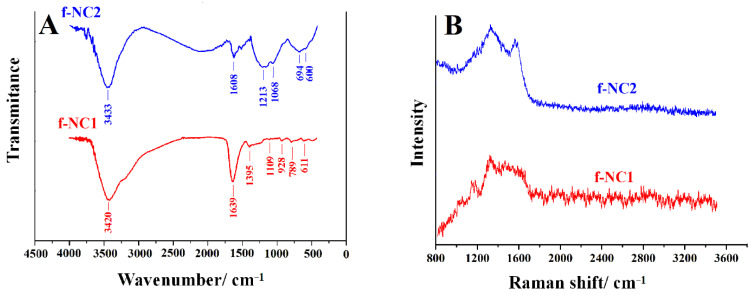
FTIR spectra (**A**) and Raman spectra (**B**) of f-NC1 and f-NC2.

The FTIR spectra of f-NC1 and f-NC2 are shown in Figure 2A. In the FTIR spectra of f-NC1, there are prominent C=N absorption peaks at about 3420 cm^−1^ and 1639 cm^−1^, indicating the formamide self-polymerisation occurred. In the FTIR spectra of f-NC2, the C=N absorption peak completely disappears. Several absorption peaks appear in the range of 1450 to 1608 cm^−1^, corresponding to the ring stretching vibration absorption peak of the pyridine and pyrrole ring. In the span of 1068–1250 cm^−1^, there is a solid and broad peak corresponding to the N-C, O-C stretching vibration absorption peak, consistent with the XRD results. The Raman spectra of f-NC1 and f-NC2 are shown in Figure 2B. The D-band peaks of f-NC1 and f-NC2 are around 1350 cm^−1^. The G-band peak around 1590 cm^−1^ of f-NC1 is inconspicuous, but the ID/IG value of f-NC2 is 1.31, which is lower than that of f-NC1, indicating a more ordered structure and a lower number of defects of f-NC2 due to the pyrolysis.

**Figure 3:**
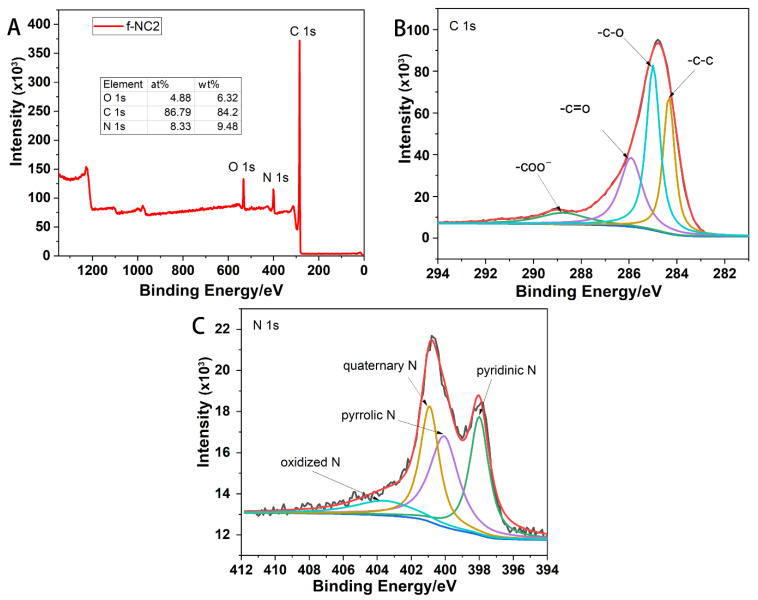
XPS spectra of the f-NC2 nanohybrid: (**A**) survey spectrum, (**B**) C 1s spectrum, (**C**) N 1s spectrum.

The elemental composition and chemical state of these materials were also investigated using XPS, as shown in Figure 3A–C. From the survey spectrum of XPS (Figure 3A), the atomic percentages of C, O and N in f-NC2 materials are 86.79%, 4.88%, and 8.33%, respectively. In addition, four characteristic peaks of C-C (284.8 eV), C-O/C-N (285.78 eV), N-C=N/C=O (287.00 eV) and COO^–^ (288.98 eV) can be seen in the high-resolution C 1s spectrum, indicating that N has been successfully doped into the carbon material (Figure 3B) [28,29]. Four characteristic peaks of pyridine N (398.1 eV), pyrrole N (399.4 eV), graphite N (400.8 eV) and oxidation state N (403.4 eV) in the high-resolution N 1s spectra (Figure 3C) were found. The XPS results above show that the synthesized N.C. material contains nitrogen and carbon elements, which further verifies the effectiveness of XRD results. Generally, the electrochemical performance of nanocomposites can be improved by doping N element in carbon nanomaterials [30,31]. Therefore, the synthesized N.C. material can effectively improve the performance of modified electrodes.

### 2.2. Electrochemical Characterisation

**Figure 4:**
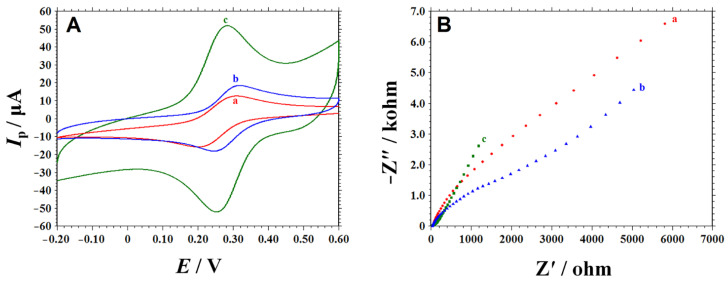
C.V.s (**A**) and EIS (**B**) of different electrodes: bare GCE (a), f-NC1/GCE (b) and f-NC2/GCE (c) in 1.0 mmol L^−1^ K_3_Fe(C.N.)_6_ solution with 1.0 mol L^−1^ KCl as supporting electrolyte. The inset in Figure 4B represents the equivalent circuit models.

The bare GCE, and the GCE modified with f-NC1 and f-NC2 (denoted as f-NC1/GCE and f-NC2/GCE) were subjected to CV (Figure 4A) and EIS (Figure 4B) characterisation in a 1.0 mM K_3_Fe(C.N.)_6_ solution containing 0.5 M KCl. Compared with the bare GCE (*I*_pa_ = 10.53 mA, *I*_pc_ = 11.95 mA) (Figure 4A, curve a), the peak current increased slightly (*I*_pa_ = 14.38 mA, *I*_pc_ = 14.37 mA) on the f-NC1/GCE (Figure 4A, curve b), while it increased significantly for f-NC2/GCE with *I*_pa_ of 37.58 mA and *I*_pc_ of 37.27 mA (Figure 4A, curve c), showing that f-NC2 has an amplifying effect on the current of K_3_Fe(C.N.)_6_. The active electrochemical area of the electrode can be obtained from the Randles-Sevcik equation [32]:

*I_p_* = (2.69 × 10^5^) *n*^3/2^
*D*^1/2^
*A C*_0_
*v*^1/2^ (1)

where *n* is the electron transfer in the electrochemical reaction, *D* is the diffusion coefficient of the reactant (*D* is about 6.3 × 10^−6^ cm^2^ s^−1^) [32], *I_p_* represents the peak current, *v* is the scan rate, and *C*_0_ is the concentration of the reactant. As shown above, the active electrochemical areas (*A*) of GCE, f-NC1/GCE and f-NC2/GCE were calculated to be 0.056 cm^2^, 0.067 cm^2^ and 0.175 cm^2^, respectively. The results indicated that the electrochemically active area of the electrode could effectively be increased by modifying the f-NC2 on the electrode surface, and more reaction sites were provided for 4-NP reduction.

Figure 4B shows the Nyquist plot of various modified electrodes. The most common and representative Nyquist plot in impedance measurements includes a semicircular one at high frequencies and a linear portion at low frequencies. The electron transfer dynamic resistance (*R*_ct_) of the redox probe can be reflected by the diameter of the semicircle in the high-frequency region. Figure 4B shows that EIS has the same trend as CV characterization. The impedance of bare GCE (Figure 4B, curve a, 48.87 Ω) is relatively large. However, the impedance decreased slightly at f-NC1/GCE (Figure 4B, curve b, 32.6 Ω), while f-NC2/GCE (Figure 4B, curve c, 29 Ω) is significantly lower than those of f-NC1/GCE and bare GCE. The results above confirmed that f-NC2 has better conductivity after carbonization. This result may relate to the typical wrinkle and folded morphologies of f-NC2. It has a large specific surface area, improving electron transfer efficiency.

### 2.3. Voltammetric Behaviour of 4-NP at Different Electrodes

**Figure 5:**
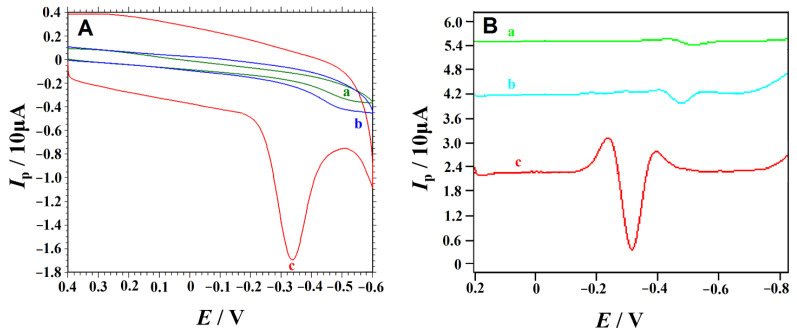
Cyclic voltammograms (**A**) and second derivative linear sweep voltammograms (**B**) of 0.1 mM 4-NP in 0.2 M H_2_SO_4_ solution obtained at bare GCE (a), f-NC1/GCE (b) and f-NC2/GCE (c). Scan rate: 0.1 V s^−1^.

Figure 5A showed the cyclic voltammograms of 0.1 mM of 4-NP in 0.2 M of H_2_SO_4_ solution obtained at bare GCE, f-NC1/GCE, and f-NC2/GCE in the potential range from 0.4 to −0.6 V at a scan rate of 0.1 V s^−1^ after accumulating at 0.3 V for 60 s. As illustrated in Figure 5A, on bare GCE, an inconspicuous cathodic peak (*I*_pc_ = 0.3369 mA) appears at −0.544 V (curve a), demonstrating that there is a high overpotential in the reduction of 4-NP at the GCE and the electron transfer rate is very slow. A slight increase in the peak current of 4-NP (*I*_pc_ = 0.5385 mA) was observed at −0.489 V on f-NC1/GCE, which may be due to the low graphitization and poor conductivity of the f-NC1 without pyrolysis treatment.

However, the peak current increased significantly when the f-NC2/GCE exhibited an enhanced electrochemical signal of 4-NP compared to those at the GCE and f-NC1/GCE (*I*_pc_ = 11.74 mA), and a more positive potential (−0.320 V) was presented. These phenomena show that f-NC2 has a strong electrocatalytic effect on 4-NP reduction, which can be attributed to the unique structure and properties of f-NC2. f-NC2 should have a strong adsorption ability for 4-NP because there are many interactions such as a hydrogen bond and hydrophobic force between them. 4-NP has an aromatic structure, so *p–p* stacking may occur between f-NC2 and 4-NP. The graphene-like structure of f-NC2 is also beneficial for increasing the specific surface area of the electrode and increasing the loading amount of 4-NP on the electrode surface. Furthermore, the conductivity of f-NC after pyrolysis is greatly increased, which is also a fundamental reason for the significant increase of 4-NP current.

Second derivative linear sweep voltammetry (SDLSV) was also performed to examine the electrochemical sensing of f-NC2/GCE towards 4-NP reduction under the same conditions. SDLSV is often used for quantitative analysis because of its small background current and high signal-to-noise ratio [33,34]. As shown in Figure 5B, poor electrochemical response of 4-NP was exhibited at the bare GCE. The f-NC1/GCE showed a better reduction peak than the bare GCE. However, a sharp cathodic peak at −0.320 V with 27.53 μA current was observed at the f-NC2/GCE. The comparison results on 4-NP sensing on different electrodes using SDLSV reveal that f-NC2/GCE significantly enhances the sensitivity of 4-NP analysis because of its good conductivity and high adsorption capacity towards 4-NP. The results are consistent with those obtained using cyclic voltammetry.

### 2.4. Optimisation of Determination Conditions

#### 2.4.1. Influences of Supporting Electrolyte and Solution pH

The influences of different supporting electrolytes, such as HCl, H_2_SO_4_, HNO_3_, H_3_PO_4_, HAc–NaAc (pH 4–6), HAc–NH_4_Ac (pH 4–6), NaH_2_PO_4_–Na_2_HPO_4_ (pH 3–6), (CH_2_)_6_N_4_–HCl (pH 4–6), Britton–Robinson (B–R) buffer (pH 3–6) (each 0.1 M), on the oxidation peak current of 4-NP were examined. Among these supporting electrolytes, the oxidation peak current of 4-NP is the highest, and the background current is the smallest in H_2_SO_4_.

**Figure 6:**
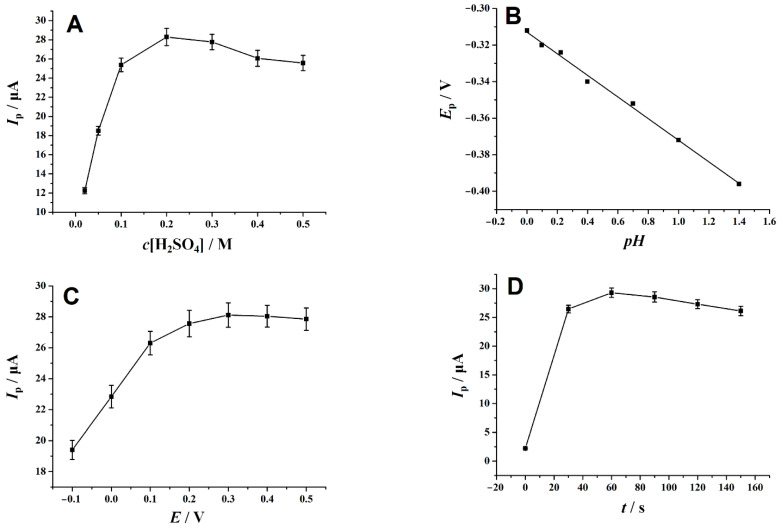
The optimum of experimental conditions: (**A**) the effect of H_2_SO_4_ concentration on the peak current, (**B**) the effect of solution pH on the peak potential, (**C**) the effect of accumulation potential on the peak current and (**D**) the effect of accumulation time on the peak current. 4-NP concentration: 0.1 mM, scan rate: 0.1 V s^−1^.

The acidity of the solution also significantly affects the electrochemical response of 4-NP. Since the determination is carried out in an H_2_SO_4_ solution, the pH value of the solution can be achieved by changing the concentration of H_2_SO_4_. As shown in Figure 6A, the peak current increases rapidly during the H_2_SO_4_ concentration from 0.02 M to 0.2 M, then decreases gradually from 0.2 M to 0.5 M. Therefore, 0.2 M was chosen for the following experiments. Figure 6B indicated that a negative shift in anodic peak potential was occurred by increasing the pH of a solution. The linear regression equation of the calibration curve was *E*_pa_ (V) = −0.05914 pH−0.3129 with a correlation coefficient of 0.9978. A slope of −0.05914 V/pH indicated that the transferred electron number was equal to the number of protons.

#### 2.4.2. Effect of Accumulation Conditions

To obtain the best performance for 4-NP detection, we studied the effects of the accumulation conditions, including accumulation potential and accumulation time, on the peak current of 4-NP. As shown in Figure 6C, the effect of accumulation potential on the electrochemical behaviour of 4-NP at f-NC2/GCE was examined over the range from −0.1 V to 0.5 V. With the positive shift of accumulation potential, the peak current of 4-NP increased and kept stable after 0.3 V. To investigate the influence of accumulation time, the peak current was detected at the accumulated potential of 0.3 V at 30 s interval time. As shown in Figure 6D, the peak intensity of 4-NP increases with the accumulation time and then tends to flatten when the accumulation time is longer than 60 s, which may be due to the saturated adsorption of 4-NP on the electrode surface. Therefore, accumulation conditions (0.3 V, 60 s) were applied in subsequent experiments.

### 2.5. Effect of Scan Rate

**Figure 7:**
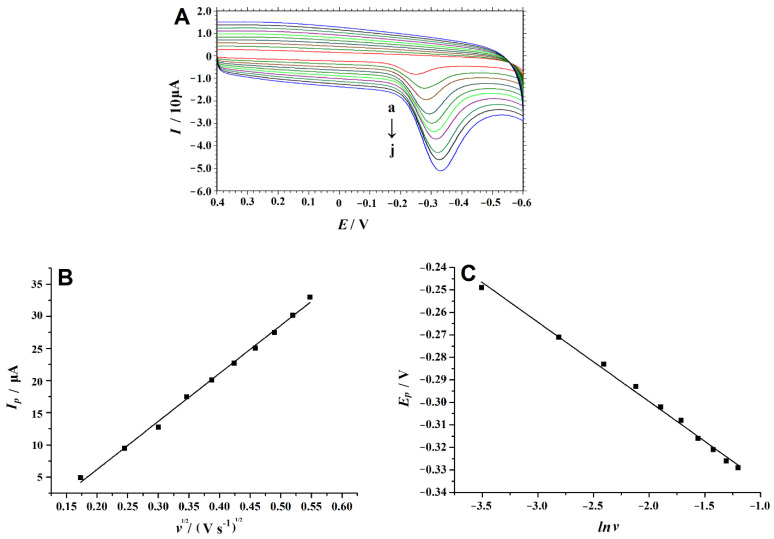
(**A**) C.V.s of 0.1 mM 4-NP at different scan rates in 0.2 M H_2_SO_4_ solution on the f-NC2/GCE (a–j: 0.03–0.30 V s^−1^, interval of 0.03 V s^−1^); (**B**) Plot of peak current vs. *v*^1/2^ for 4-NP; (**C**) Plot of peak potential vs. ln*v* for 4-NP.

**Scheme 1:**
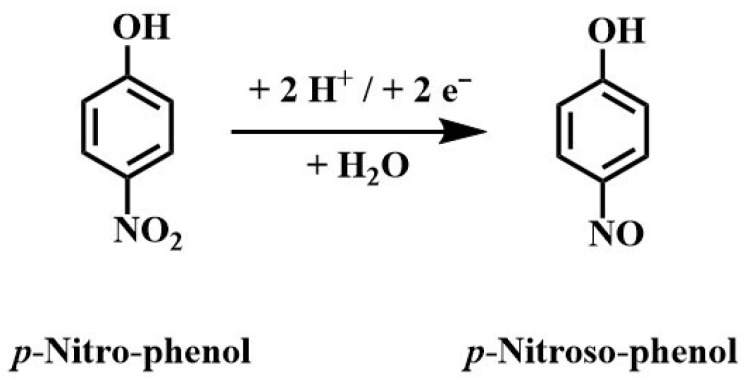
Schematic of the reduction mechanism of 4-NP on the f-NC2/GCE.

To further clarify the reaction process of 4-NP, the effect of the scan rate on the electrochemical response of 4-NP was studied by cyclic voltammetry. The superimposed voltammograms with different scan rates was presented Figure 7A. A linear relationship between the *I*_p_ and *v*^1/2^ (Figure 7B): *I*_p_ = 74.643*v*^1/2^ − 8.7352 (R = 0.9981) indicated a diffusion-controlled electrode process 4-NP. Similarly, the linear relationship between *E*_p_ and ln*v* (Figure 7C) was expressed as an equation: *E*_p_(V) = −0.0343ln*v* − 0.3703 (R^2^ = 0.9952). According to Laviron’s theory [35] and R.T./αnF = 0.0343, the number of electrons transferred (*n*) was calculated to be about two as the value of the charge transfer coefficient (α) was assumed as 0.5 for an irreversible electrode reaction process. Since the previous conclusion is that the number of protons and electrons involved in the reaction is equal, there are two electrons and two protons in the reduction process of 4-NP, which is consistent with the previous result [36]. The plausible mechanism of 4-NP is shown Scheme 1.

### 2.6. Chronocoulometric Curve

**Figure 8:**
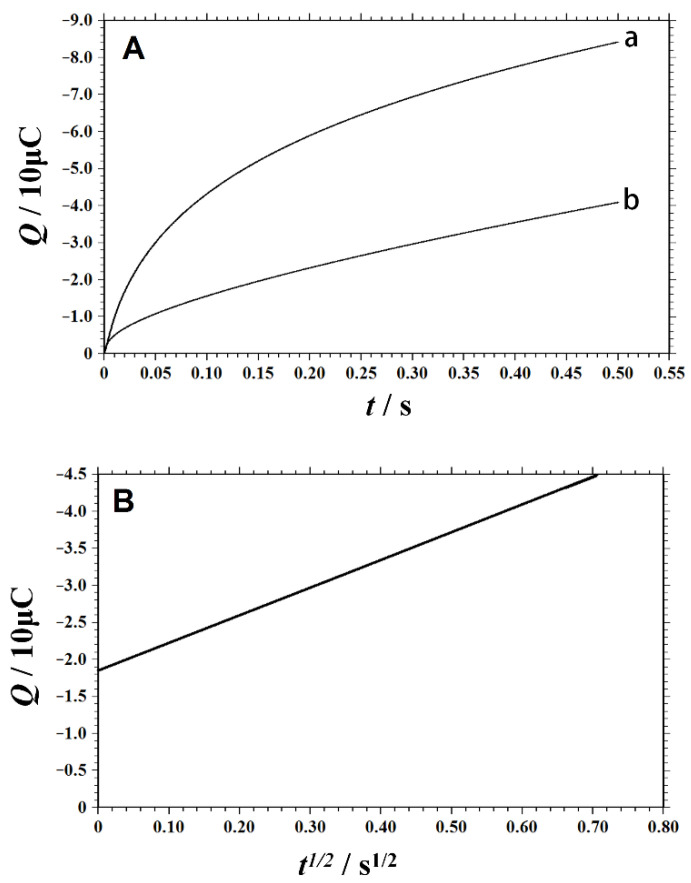
(**A**) Plot of *Q*–*t* curve of f-NC2/GCE in 0.2 M H_2_SO_4_ solution containing 0.1 mM 4-NP (curve a). plot of *Q*–*t*^1/2^ curve on the f-NC2/GCE (background subtracted, curve b). (**B**) stand for that in the blank solution.

Since 4-NP is a diffusion-controlled reaction process on the f-NC2/GCE, the diffusion coefficient (*D*) of 4-NP in a solution can be calculated through the chronocoulometric curves of 4-NP on the modified electrode. Figure 8A showed the corresponding curves obtained on the f-NC2/GCE in a given solution (curves a and b), and Figure 8B showed a good linear relationship between *Q* and *t*^1/2^. The linear regression equation can be expressed as *Q* (μC) = 37.36*t*^1/2^ + 18.43 (R = 0.9891), based on Anson’s equation [37]:

*Q* = 2*nFAcD*^1^^/2^p − 1/2*t*^1/2^ + *Qdl + Qads* (2)

The *D* of 4-NP was calculated as 9.6 × 10^−5^ cm^2^ s^−1^. For the completely irreversible reduction of 4-NP at the f-NC2/GCE, the standard heterogeneous rate constant (*k*_s_) can be obtained according to Equation (3) [38]:

*k*_s_ = 2.415 exp (−0.02*F/RT*)*D*^1/2^(*E*_p_ − *E*_p/2_) − 1/2*v*^1/2^ (3)

In the formula, *E*_p/2_ is the potential at which *I* = *I*_p/2_, *E*p represents the peak potential, and other symbols have their usual meanings. In our experiment, *E*_p_ − *E*_p/2_ = 58 mV, *D* = 9.6 × 10^−5^ cm^2^ s^−1^, *v* = 100 mV s^−1^, and T = 298 K. The value of *k*_s_ was calculated to be 1.429 × 10^−3^ cm s^−1^. It shows that the reaction of 4-NP on the electrode is a very rapid process.

### 2.7. Evaluation of Electrode Performance

#### 2.7.1. Selectivity

In electrochemical detection, the performance of the electrochemical sensor will be suppressed by the interference of some accompanying substances in the samples. We use the SDLSV technique to study the anti-interference performance of the sensor. In 0.2 M of H_2_SO_4_ solution containing 10 μM of 4-NP, some phenolic organic compounds of 10-fold concentration were added, such as phenol, hydroquinone, catechol, resorcinol, 2-chlorophenol, 2,4-dichlorophenol, 2,4,6-trichlorophenol, 2-aminophenol, 4-aminophenol, 2-nitrophenol (2-NP) and 3-nitrophenol (3-NP). The experimental results showed that the current response of 4-NP changed little in the presence of these interferences (the signal change was less than ±5.0%). At the same time, the influence of common inorganic ions on the determination was also investigated. In the presence of 100 times the excess concentration of Cu^2+^, Co^2+^, Zn^2+^, Ca^2+^, Mg^2+^, Pb^2+^, Cd^2+^, K^+^, Al^3+^, Fe^3+^, Ni^2+^, Cr^3+^, Mn^2+^, Cl^−^, NO_3_^−^ and SO_4_^2−^, the influence of peak current response of 4-NP can be ignored (the signal change was less than ±5.0%). These results demonstrated that the sensor has good selectivity in detecting 4-NP.

#### 2.7.2. Reproducibility, Stability and Repeatability

Excellent reproducibility, stability and repeatability are the remarkable characteristics of high-efficiency electrochemical sensors. Therefore, it is essential to evaluate the prepared sensor’s reproducibility, stability, and reproducibility. The same procedure fabricated eight identical f-NC2/GCEs. We measured the peak currents obtained on these f-NC2/GCEs in the presence of 10 mM of 4-NP. The results showed that the current responses received on the eight electrodes were almost the same, with no significant difference. The RSD was 4.17%, indicating that the f-NC2/GCE has good reproducibility. The repeatability of the f-NC2/GCE was also evaluated by the same modified electrode to measure the cathodic peak current of 10 mM of 4-NP for ten consecutive times. The RSD was only 2.20%, which shows the excellent repeatability of the sensor. We stored the electrode in the air at room temperature, and its long-term stability was estimated by intermittent determination of 4-NP. After two weeks, we discovered that the peak current kept about 93.57% of its initial value. After storage for a month, the peak current remained approximately 86.64% of its initial value, showing outstanding stability. This may be due to the excellent stability of f-NC2.

#### 2.7.3. Linear Range and Detection Limit

**Figure 9:**
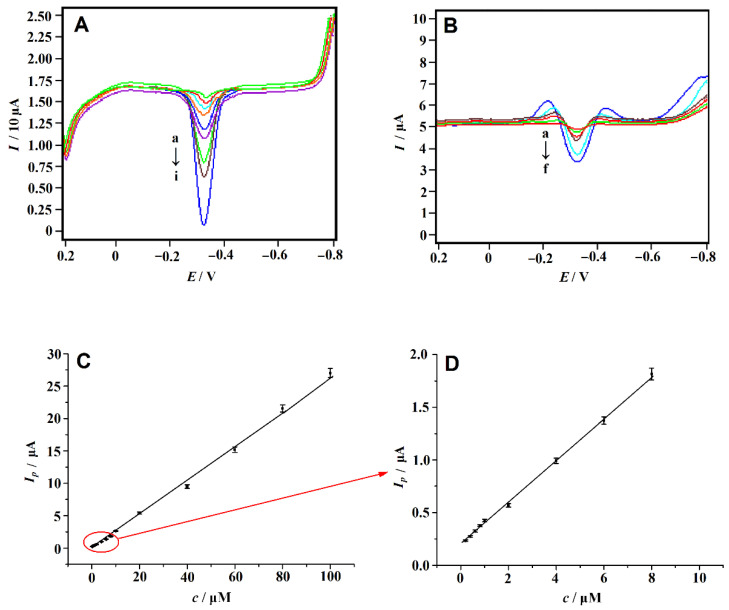
The curves of SDLSV of different concentration 4-NP in 0.2 M H_2_SO_4_ solution obtained at the f-NC2/GCE at a scan rate of 0.1 V s^−1^ after accumulation at 0.3 V for 60 s. (**A**) a–i: 0.2, 0.4, 0.6, 0.8, 1.0, 2.0, 4.0, 6.0, 8.0 μM; (**B**) a–f: 10, 20, 40, 60, 80, 100 μM. (**C**) The linear relationship between the *I*_p_ and 4-NP concentration in range of 0.2 to 100 μM. (**D**) Calibration curve for 4-NP in low concentration range (0.2–8.0 μM).

The SDLSV technique was used to determine 4-NP on the f-NC2/GCE at different concentrations in this experiment. The resultant SDLSV curves obtained are shown in Figure 9A,B, The cathodic peak current increased with the increase of 4-NP concentration. Figure 9C,D show the linear correlation between current and concentration and the corresponding calibration curves. In the linear range of 0.2 to 100 μM, the linear regression equation was *I*p (μA) = 0.2652 *c*(μM) − 0.0443, and the correlation coefficient was 0.9988. The limit of detection (LOD) was as low as 0.02 μM, and good sensitivity of 1.518 μA μM^−1^ cm^−2^ was obtained, indicating that the developed f-NC2/GCE has excellent performance for 4-NP detection.

In addition, we compared the performance of f-NC2/GCE with other modified electrodes according to the LOD and linear range, as shown in Table 1. The LOD obtained by f-NC2/GCE was better than most reported modified electrodes but not as good as graphene-modified acetylene black paste electrode [39], NiO nanoparticles and *N*-hexyl-3-methylimidazolium hexafluorophosphate modified carbon paste electrode [16], and carbon nanofiber decorated with samarium (III) oxide nanoparticles modified screen-printed carbon electrode [19]. However, the f-NC2/GCE has the advantages of low cost, simple preparation, a fast response time (60 s), good reproducibility and stability. Therefore, the voltammetric determination of 4-NP is easily interfered with by other reducible compounds. In this study, 4-NP produced a sharp reduction peak at −0.320 V on the f-NC2/GCE, which was more positive than other electrodes. These results indicate that f-NC2 is an ideal material for sensors, and f-NC2/GCE can detect 4-NP with high sensitivity and efficiency.

#### 2.7.4. Sample Analysis

The technique of SDLSV was used to analyse various water samples of river water, spring water, lake water, tap water and domestic sewage without any pretreatment under the above optimal conditions. This also helped to verify the feasibility of 4-NP in environmental water samples. No electrochemical response of 4-NP was found in all the samples, which may be due to the absence of 4-NP in the water samples or the concentration of 4-NP was lower than the method’s detection limit. Therefore, the standard addition method applies the process to determine 4-NP in samples spiked with 4-NP at a specific concentration. The recoveries of 4-NP in these water samples were calculated, and the recovery values ranged from 97.4% to 104.0%. Therefore, the sensor constructed in this study is suitable for the quantitative analysis of 4-NP in actual water samples.

## 3. Materials and Methods

### 3.1. Reagents

Formamide (F.A.) and 4-nitrophenol (4-NP) were obtained from Sinopharm Chemical Reagent Co., Ltd., Shanghai, China. 0.2 M of H_2_SO_4_ solution were used as a supporting electrolyte. All reagents were of analytical grade. Ultrapure water (resistivity > 18 MW cm) was obtained from a Milli-Q Plus system (Millipore, Paris, France).

### 3.2. Apparatus

X-ray diffraction (XRD) patterns were obtained from a powder X-ray diffractometer (D8 Advance, Bruker AXS GmbH, Karlsruhe, Germany) with Cu Ka radiation (l = 0.154056 nm). Scanning electron microscopy (SEM) images were received from a scanning electron microscope (EVO10, ZEISS, Jena, Germany) operated at an acceleration voltage of 20.0 kV. Raman spectra were performed on a LabRAM Aramis Raman spectrometer (HORIBA Jobin Yvon, Paris, France). Transmission electron microscope (TEM) images were recorded with a transmission electron microscope (JEOL JEM-2100, Tokyo, Japan). X-ray photoelectron spectroscopy (XPS) was obtained on a Thermo Electron ESCALAB250 XPS Spectrometer (X-ray Source: Al). Fourier transform infrared spectroscopy (FTIR) spectra were recorded on an IRPrestige-21 Fourier transform infrared spectrometer (Shimadzu Corp., Kyoto, Japan). A traditional three-electrode system (unmodified or modified GCE (id = 3.0 mm)) was used as the working electrode, platinum wire as the counter electrode and saturated calomel electrode (SCE) as the reference electrode for all electrochemical tests. Second derivative linear sweep voltammetry (SDLSV) was performed on a JP-303E polarographic analyzer (Chengdu instrument factory, Chengdu, China).

### 3.3. Synthesis of f-NC Material

The preparation of f-NC material was carried out according to the following steps: Transference of 30 mL formamide to a 40 mL Teflon-lined autoclave, sealed and heated at 180 °C for 12 h. The obtained brown-black product was centrifuged, washed with water and ethanol, and then dried in a vacuum at 60 °C overnight. The resulting product (expressed as f-NC1) was placed in a corundum boat with a cover and the tubular furnace was set to the following temperature procedure: first, under nitrogen atmosphere, the temperature was raised to a heating rate of 5 °C min^−1^ from 25 °C to 400 °C, and kept at 400 °C for two h. The temperature was then increased to 900 °C at a heating rate of 10 °C min^−1^ and was held at 900 °C for two h to carbonise the material thoroughly. Finally, the temperature was reduced to room temperature to obtain the final product (expressed as f-NC2).

### 3.4. Electrode Preparation and Modification

The bare glassy carbon electrode (GCE) was polished with 0.05 μm of alumina suspension, then sonicated in ultrapure water and ethanol for 1 min in turn and finally dried under an infrared lamp. To obtain a smooth and mirror-like shape before modification, 5.0 mg of f-NC1 and f-NC2 were added into 5 mL of ultrapure water each. The two suspensions were sonicated for 20 min to obtain a uniform dispersion. 5.0 μL of the above dispersions were dropped onto the surface of GCE with a micro syringe and then dried at room temperature to prepare the modified electrodes. The modified electrodes obtained were labelled as f-NC1/GCE and f-NC2/GCE, respectively.

### 3.5. Procedure of Voltammetric Measurements

We added the standard solution containing 4-NP of appropriate concentration into the electrolytic cell containing 10 mL of H_2_SO_4_ solution (0.2 M), and the three-electrode system was immersed in the above test solution. After stirring at a potential of 0.3 V for 60 s and standing still for 5 s, the cyclic voltammogram or second derivative linear scanning voltammogram was recorded between 0.2 V and −0.8 V at a scanning rate of 0.1 V s^−1^. After each voltammetric determination, to remove the measured substance on the electrode surface, the modified electrode was put into 0.2 M of H_2_SO_4_ solution for continuous cyclic voltammetric scanning until there was no peak and the curve was stable. This treatment can eliminate the memory effect of the electrode and obtain a stable electrochemical response. The same procedure was carried out in the water sample analysis, and all electrochemical experiments were performed at room temperature.

## 4. Conclusions

To summarise, a novel electrochemical sensor using a nitrogen carbon material based on condensation and carbonisation of formamide (denoted as f-NC) was successfully constructed. A susceptible and reproducible 4-nitrophenol sensing platform was fabricated by coating f-NC on the glassy carbon electrode. The f-NC effectively catalysed the reduction of 4-nitrophenol on the electrode surface because of its good conductivity and excellent adsorption and enrichment ability for 4-NP. The platform was applied to determine 4-NP in environmental water samples. The results show that the f-NC is a promising material for preparing electrochemical sensors.

## Figures and Tables

**Table 1:** Performance comparison of the f-NC2/GCE with other electrochemical sensors for 4-NP detection.

Technique	Sensor	Linear Range (μM)	Detection Limit (μM)	Fabrication Reproducibility/RSD%	Stability Keep	Reference
^a^ SDLSV	^f^ GR/ABPE	0.02–8.0; 8.0–100	0.008	4.2	87% (30 days)	[11]
^b^ LSV	^g^ GO/GCE	0.1–120	0.02	3.25	83% (30 days)	[12]
LSV	^h^ PDDA-G/GCE	0.06–110	0.02	3.43	95.4% (14 days)	[13]
^c^ CV	^i^ MIP-PANI/GO/CPE	60–1400	20	-	-	[14]
CV	^j^ PCZ@N-GE/GCE	0.8–20	0.062	3.5	90.0% (5 days)	[15]
^d^ SWV	^k^ NiONPs-NH_3_MPF_6_/CPE	0.01–280	0.007	3.7	92.5% (20 days)	[16]
Amperometry	^l^ MWCNTs-MnO_2_/GCE	30–475	0.64	3.17	96.2% (1500 s)	[17]
^e^ DPV	^m^ AgPd@UiO-66-NH_2_/GCE	100–370	0.032	-	-	[18]
DPV	^n^ Sm_2_O_3_ NPs@*f*-CNFs/SPCE	0.02–387.2	0.00261	-	-	[19]
SDLSV	^o^ f-NC2/GCE	0.2–100	0.02	4.17	86.64% (30 days)	This work

^a^ second-order derivative linear sweep voltammetry; ^b^ Linear sweep voltammetry; ^c^ cyclic voltammetry; ^d^ square wave voltammetry; ^e^ differential pulse voltammetry; ^f^ graphene modified acetylene black paste electrode; ^g^ graphene oxide modified glassy carbon electrode; ^h^ poly(diallyldimethylammonium chloride) functionalized graphene modified glassy carbon electrode; ^i^ molecularly imprinted polyaniline/graphene oxide composite modified carbon paste electrode; ^j^ Polycarbazole/nitrogen-doped graphene composite modified glass carbon electrode; ^k^ NiO nanoparticles and N-hexyl-3-methylimidazolium hexafluorophosphate modified carbon paste electrode; ^l^ multi-walled carbon nanotube/manganese dioxide nanocomposite modified glassy carbon electrode; ^m^ silver and palladium bimetallic nanoparticles functionalized metal organic frameworks modified glassy carbon electrode; ^n^ carbon nanofiber decorated with samarium (III) oxide nanoparticles modified screen-printed carbon electrode; ^o^ formamide-converted nitrogen-carbon materials modified glassy carbon electrode.

## Data Availability

Date are available as requested.
